# Effects of disability-related limitations in daily living on unmet needs: a longitudinal-study^1^

**DOI:** 10.1186/s12889-024-17674-z

**Published:** 2024-02-02

**Authors:** Hanseul Kim, Yun Hwa Jung, Sohee Park, Jaeyong Shin, Sung-In Jang

**Affiliations:** 1 Southern Seoul Regional Headquarters, National Pension Service, Seoul, Republic of Korea; 2 Department of Public Health, Graduate School, Yonsei University, Seoul, Republic of Korea; 3 Institute of Health Services Research, Yonsei University, Seoul, Republic of Korea; 4 Department of Biostatistics, Graduate School of Public Health, Yonsei University, Seoul, Republic of Korea; 5 Department of Preventive Medicine & Institute of Health Services Research, Yonsei University College of Medicine, 50 Yonsei-ro, Seodaemun-gu, 03722 Seoul, Republic of Korea

**Keywords:** People with disabilities, Unmet needs, Health services needs and demand, Daily living limitations, Activities of daily living

## Abstract

**Background:**

Unmet health needs are particularly important to people with disabilities; however, these unmet needs owing to limitations in daily life have been under-researched thus far. This study examined the effects of disability-related limitations in daily life on unmet needs.

**Methods:**

This study included 5,074 adults with disabilities from the 2018–2020 Korea Disability and Life Dynamics Panel. We analyzed the effects of disability-related limitations in daily life on unmet needs using logistic regression with a generalized estimating equation model.

**Results:**

Overall, 4.8% men and 4.6% women with disabilities had unmet needs. For men, unmet needs were 1.46 times (95% confidence interval [CI] 1.09–1.96) higher for those with moderate limitations in daily life. For women, unmet needs were 1.79 times (95% CI 1.22–2.39) higher when there were moderate limitations in daily life. The prominent factors causing this effect were physical or brain lesion disability for men and internal or facial disability and burden of medical expenses for women.

**Conclusions:**

Limitations in daily life due to disability increase the risk of having unmet needs, an effect that is significantly more pronounced in men. These unmet needs differ depending on an individual’s sex, disability type, limited body parts, and other specific causes. Efforts are required to reduce the unmet needs of people with disabilities by considering the type of disability, impaired body parts, and causes of unmet needs in daily life.

## Introduction

Although access to medical care has gradually improved, unmet health needs still remain an important public health concern. Unmet health needs are defined as the medical gap between perceived or assessed needs and the actual resources available [1]. Unmet health needs harm independence, overall health, and quality of life from an individual’s perspective [2–4] and give rise to health inequalities from a social perspective. Health-vulnerable groups are more sensitive to the adverse effects of these unmet needs.

According to prior research, disability increases an individual’s odds of not receiving necessary routine care by more than 50% [5]. Individuals with disabilities have three times more unmet needs than those without disabilities [6]. Studies have found that the prevalence of disability is 35.2% in Finland, 27.3% in the United Kingdom, 12.7% in the United States, and 7.6% in Japan [7, 8]. Since the global population of people with a disability is estimated to be 15.6% [9], their unmet needs constitute a significant portion of the population dealing with health inequality.

In 2020 in South Korea, the prevalence of disabilities was 5.1%, of which 87.9% represented individuals who had acquired disabilities [10]. Additionally, in South Korea, single-person households accounted for the largest share of household types at 27.9% in 2017; as per a prior study the proportion of aging people with disabilities (51.3%) will reach three times that of the total population in 2021 [8]. Put differently, concerning situations like an increased risk of acquired disabilities due to longer lifespans, and an aging population can lead to an increase in the potential risk group for limitations in daily life due to disabilities.

Individuals with disabilities experience both physical and mental limitations in their daily lives. Therefore, they may have varying degrees of unmet needs compared to individuals without disabilities. Nonetheless, previous studies on unmet medical care for people with disabilities were limited to specific types of disabilities or diseases, or have focused on functional performance, such as daily life or instrumental activities of daily living [11–14].

Therefore, identifying unmet needs based on the areas and degrees of everyday limitations caused by physical or mental disabilities can help fill this knowledge gap. This study aims to provide evidence on the association between daily living limitations due to disability and unmet needs. This study primarily focused on analyzing the unmet needs according to the degree of limitation; however, it also analyzed specific physical or mental limitations and types of disabilities and causes of unmet needs. This study hypothesized that increased limitations of daily activities due to disability increase the risk of having unmet needs.

## Methods

### Data

This study used Disability and Life Dynamics Panel (DLDP) data from waves 1–3 (2018–2020), as surveyed by the Korea Disabled People’s Development Institute (KODDI). DLDP is a comprehensive nationwide dataset designed to assess changes in various aspects of life after individuals register for disability in the country. This longitudinal time-series survey was double-sampled with stratified variables for disability type, degree of disability, and sex, based on the Registered Disabled Persons Register of the Ministry of Health and Welfare of Korea (National approved statistics number: 438,001). The final panel consisted of 6,121 individuals with disabilities, and the relative standard error, calculated using weights, was found to be less than 5% at the national level. The survey was conducted with the consent of the participants by a professional interviewer, with a focus on four major areas (acceptance of and change in disability, health and medical care, self-reliance, and social participation), through face-to-face tablet-assisted personal interviews. In cases where the respondents were unable to communicate due to physical or mental disabilities, conditional proxy responses were allowed from guardians or authorized representatives. The DLDP dataset comprises de-identified secondary public data [15]. This study was approved for review exemption by the Institutional Review Board of the Yonsei University Health System Severance Hospital (Assignment approval number: 4-2022-1374).

### Participants

The DLDP had 6,121 respondents at baseline, of whom 1,047 aged under 19 years were excluded. Response rates were 90.3% and 95.2% in the second and third waves, respectively. The final number of participants aged 19 years or older per wave were as follows: first wave: 5,074 (2,676 men, 2,307 women); second wave: 4,610; and third wave: 4,401.

### Variables

The dependent variable in this study was unmet needs. Having unmet needs was categorized as either “yes” and “no” according to an individual’s response to the question “During the past six months, were you in the need of a medical treatment at a hospital or clinic (excluding dental clinics) but did not receive it?”

The independent variable was disability-related limitations in daily living. Disability-related limitations in daily living were categorized into “low (0–13 points),” “mid (14–27 points),” and “high (28–39 points)” by dividing into three equal parts based on the totaled scores of 14 items, each measured on a 4-point Likert scale. These 14 items were created based on the Disability Identification Module used in Australia’s Survey of Disability, Aging, and Carers. This questionnaire is divided into six categories: “sensory,” “intellectual,” “physical,” “psychosocial,” “head injury, stroke or acquired brain injury,” and “other.”

The covariates were demographic (sex, age, and region), socioeconomic (marital status, education level, and household income), and health behavioral factors (drinking, smoking, and exercise). The other covariates included chronic diseases related to health status, as well as disability type and level. The type and level of disability were determined according to Korean disability classification criteria and KODDI’s panel survey design. Chronic diseases include diseases that affect daily life, including cancer, diabetes, high blood pressure, chronic renal failure, arthritis, mental illness, etc.

### Statistical analysis

**Fig. 1:**
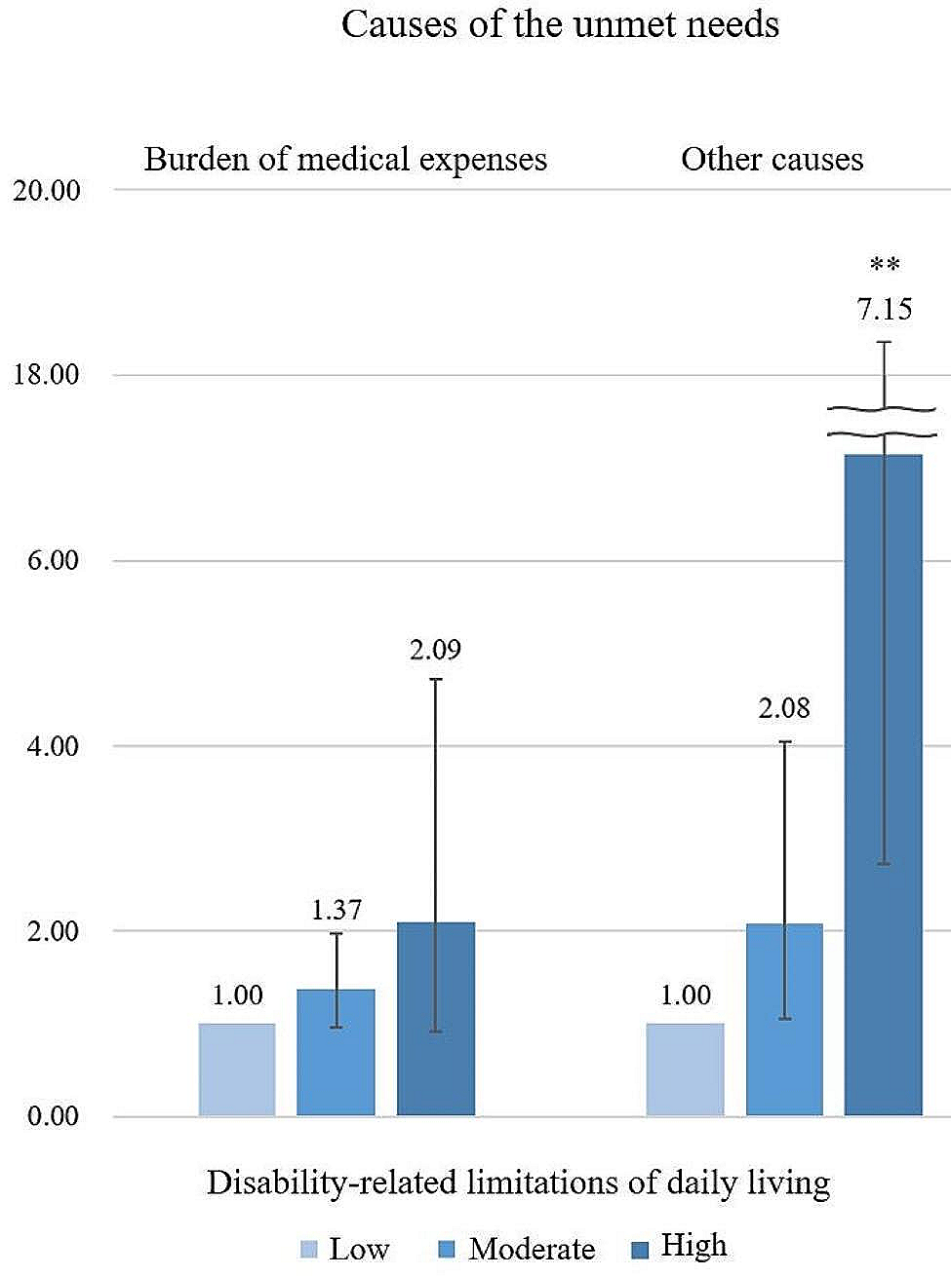
Contents of the effect of disability-related limitations of daily living on the causes of the unmet needs in men. ^a^ Adjusted for other covariates. Reference group: Low disability-related limitations of daily living. Statistically significant *: *p* ≤ 0.05, **: *p* ≤ 0.01, ***: *p* ≤ 0.001, ****: *p* ≤ 0.0001

**Fig. 2:**
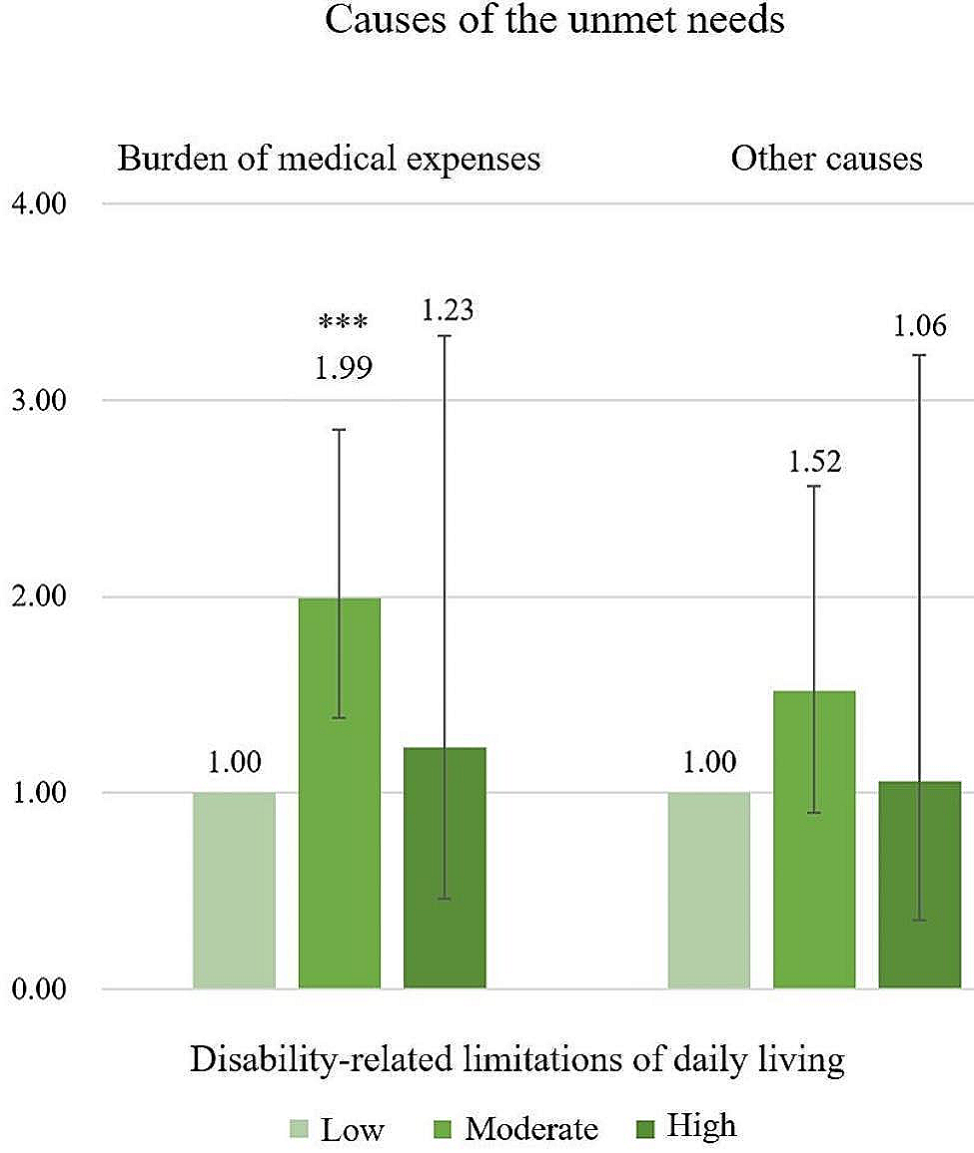
Contents of the effect of disability-related limitations of daily living on the causes of the unmet needs in women. ^a^ Adjusted for other covariates. Reference group: Low disability-related limitations of daily living. Statistically significant *: *p* ≤ 0.05, **: *p* ≤ 0.01, ***: *p* ≤ 0.001, ****: *p* ≤ 0.0001

Descriptive statistical analysis including chi-squared test were conducted to investigate the frequency and distribution of the study participants’ general characteristics in Tables 1 and 2. For the main and sub-analyses, the generalized estimating equation model (GEE) was used to determine the effect of daily limitations due to disability on unmet needs. The results of the main analysis conducted in this study to provide evidence for the relationship between disability-related limitations in daily living and unmet needs are presented in Table 3. Sub-analyses were performed on covariates closely related to the main association, sub-question items of the independent variable indicators, and factors of the dependent variable. A subgroup analysis was conducted and stratified according to the type and level of disability (Table 4). Sub-analyses were also conducted on the sub-questions to determine limitations in daily life due to disability (Table 5), and factors contributing to the existence of unmet health needs by sex (Figs. 1 and 2). All analyses using GEE are presented as adjusted odds ratios (AOR) with 95% confidence intervals (95% CI). In our statistical analyses, a *p*-value of less than 0.5 was considered to be statistically significant, and SAS version 9.4 (SAS Institute Inc; Cary, NC, USA) was used.

## Results

Table 1 shows the general characteristics of study participants at baseline. Among the 5,074 participants, 2,767 (54.5%) were men and 2,307 (45.5%) were women. In terms of age, the proportion of elderly people was high (19–29 years old: 6.1%; 30s: 7.2%; 40s: 13.7%; 50s: 32.6%; 60s or older: 40.4%). Approximately, three out of five participants had chronic diseases (3,089/5,074). The low and high disability severity groups were similar in size (Disability level, low: 2,490, 49.1%; high: 2,584, 50.9%).

**Table 1:** General characteristics of the study population at baseline

Variables	General characteristics of the study population		
	*N*	%	
**Sex**			
Men	2,767	54.5	
Women	2,307	45.5	
**Disability-related limitations of daily living**			
Low	3,055	60.2	
Mid	1,774	35.0	
High	245	4.8	
**Age**			
19–29	311	6.1	
30–39	363	7.2	
40–49	696	13.7	
50–59	1,654	32.6	
≥ 60	2,050	40.4	
**Region**			
Urban area	4,737	93.4	
Rural area	337	6.6	
**Marital Status**			
Living with spouse	2,787	54.9	
Single, separated, divorced, bereavement	2,287	45.1	
**Educational level**			
College or over	923	18.2	
High school	2,160	42.6	
Middle school or less	1,991	39.2	
**Household income**			
High	1,653	32.6	
Middle	1,740	34.3	
Low	1,681	33.1	
**Chronic disease**			
No	1,985	39.1	
Yes	3,089	60.9	
**Social care services**			
Not use	4,751	93.6	
Use	323	6.4	
**Drinking**			
Never	2,049	40.4	
Past	1,926	38.0	
Current	1,099	21.7	
**Smoking**			
Never	3,051	60.1	
Past	1,407	27.7	
Current	616	12.1	
**Exercise**			
Much	2,458	48.4	
Less	232	4.6	
No	2,384	47.0	
**Disability type**			
Physical disability	867	17.1	
Brain lesion disability	811	16.0	
Visual disability	667	13.1	
Hearing or language disability	835	16.5	
Intellectual or autism disability	270	5.3	
Mental disability	321	6.3	
Internal or facial disability	1,303	25.7	
**Disability level**			
Low	2,490	49.1	
High	2,584	50.9	
**Total (** *N* ** = 5,074)**	5,074	100.0	

Table 2 presents the results of descriptive statistics analysis of unmet needs by disability-related limitations of daily living. Among those with disabilities, 4.8% (133/2,767) of men and 4.6% (105/2,307) of women had unmet needs. Among men, the proportion of unmet needs gradually increased with an increase in daily life limitations (level of disability-related limitations in daily living: low, 4.0%; mid, 5.8%; high, 8.1%). Among women, the proportion of individuals with unmet needs decreased when disability-related limitations in daily living were high; the proportion of individuals with unmet needs in this high group was 3 out of 109 (level of disability-related limitations in daily living: low, 3.6%; mid, 6.5%; high, 2.8%).

**Table 2:** Results of descriptive statistical analysis of unmet needs by disability-related limitations of daily living at baseline

**Variables**	**Unmet needs**						
	**Men**						
	**Total**		**No**		**Yes**		***P-value***
	***N***	**%**	***N***	**%**	***N***	**%**	
**Disability-related limitations of daily living**							0.0276
Low	1,660	60.0	1,594	96.0	66	4.0	
Mid	971	35.1	915	94.2	56	5.8	
High	136	4.9	125	91.9	11	8.1	
**Total**	2,767	100.0	2,634	95.2	133	4.8	
**Variables**	**Unmet needs**						
	**Women**						
	**Total**		**No**		**Yes**		***P-value***
	***N***	**%**	***N***	**%**	***N***	**%**	
**Disability-related limitations of daily living**							0.0059
Low	1,395	50.4	1,345	96.4	50	3.6	
Mid	803	29.0	751	93.5	52	6.5	
High	109	3.9	106	97.2	3	2.8	
**Total**	2,307	100.0	2,202	95.4	105	4.6	

In the main analysis of Table 3, it was noted that men with medium and high levels of disability-related limitations in daily living had 1.46 times (95% CI 1.09–1.96) and 2.79 times (95% CI 1.57–4.94) as many unmet needs compared to men with low levels of unmet health needs, respectively. Unmet needs were 1.79 times (95% CI 1.22–2.39) higher in women with mid-levels of disability-related limitations in daily living than in women with low levels; however, this was not statistically significant in women with high levels of unmet health needs (AOR 1.58, 95% CI 0.80–3.12).

**Table 3:** Results of factors associated with unmet needs

Variables	Unmet needs							
	Men				Women			
	OR	95% CI			OR	95% CI		
**Disability-related limitations of daily living**								
Low	1.00				1.00			
Mid	1.46	(1.09	–	1.96)	1.79	(1.33	–	2.39)
High	2.79	(1.57	–	4.94)	1.58	(0.80	–	3.12)
**Age**								
19–29	1.00				1.00			
30–39	1.15	(0.51	–	2.57)	0.53	(0.20	–	1.38)
40–49	0.88	(0.43	–	1.81)	0.63	(0.27	–	1.46)
50–59	0.86	(0.44	–	1.72)	0.77	(0.35	–	1.71)
≥ 60	0.84	(0.40	–	1.74)	0.60	(0.27	–	1.32)
**Region**								
Urban area	1.00				1.00			
Rural area	1.22	(0.72	–	2.07)	1.19	(0.68	–	2.07)
**Marital Status**								
Living with spouse	1.00				1.00			
Single, separated, divorced, bereavement	1.50	(1.08	–	2.09)	1.17	(0.88	–	1.57)
**Educational level**								
College or over	1.00				1.00			
High school	1.07	(0.71	–	1.61)	0.90	(0.56	–	1.44)
Middle school or less	1.29	(0.83	–	2.02)	0.92	(0.57	–	1.48)
**Household income**								
High	1.00				1.00			
Middle	1.23	(0.82	–	1.85)	1.26	(0.88	–	1.82)
Low	2.22	(1.44	–	3.43)	1.53	(1.05	–	2.23)
**Chronic disease**								
No	1.00				1.00			
Yes	1.93	(1.43	–	2.62)	2.30	(1.63	–	3.24)
**Social care services**								
Not use	1.00				1.00			
Use	1.16	(0.72	–	1.86)	1.16	(0.71	–	1.89)
**Drinking**								
Never	1.00				1.00			
Past	1.10	(0.70	–	1.73)	1.16	(0.85	–	1.58)
Current	1.07	(0.65	–	1.78)	1.50	(0.93	–	2.42)
**Smoking**								
Never	1.00				1.00			
Past	0.99	(0.66	–	1.47)	1.19	(0.73	–	1.95)
Current	1.57	(1.01	–	2.44)	2.55	(1.34	–	4.86)
**Exercise**								
Much	1.00				1.00			
Less	0.98	(0.57	–	1.69)	0.85	(0.45	–	1.61)
No	1.20	(0.94	–	1.54)	1.36	(1.04	–	1.78)
**Disability type**								
Physical disability	1.00				1.00			
Brain lesion disability	0.67	(0.41	–	1.10)	0.98	(0.59	–	1.63)
Visual disability	1.22	(0.77	–	1.94)	1.71	(1.05	–	2.80)
Hearing or language disability	0.63	(0.39	–	1.04)	1.10	(0.66	–	1.84)
Intellectual or autism disability	0.73	(0.33	–	1.64)	0.94	(0.42	–	2.10)
Mental disability	0.54	(0.25	–	1.16)	0.67	(0.31	–	1.46)
Internal or facial disability	0.80	(0.52	–	1.23)	0.80	(0.49	–	1.32)
**Disability level**								
Low	1.00				1.00			
High	0.78	(0.58	–	1.05)	0.80	(0.57	–	1.11)

Disability type was stratified as a sub-analysis in Table 4. Among men, there was a dose-response tendency for disability-related limitations in daily living and unmet needs, and a large effect size was statistically significant for physical or brain lesion disability (disability-related limitations in daily living, high: AOR 2.77, 95% CI 1.22–6.29). Conversely, among women, disability-related limitations in daily living and unmet needs showed an inverse U-shape, and the effect size was prominent for internal or facial disabilities (disability-related limitations in daily living, high: AOR 7.45, 95% CI 1.11–50.00).

**Table 4:** The results of subgroup analysis stratified by independent variables

**Variables**	**Unmet needs**								
	**Men**								
	**Disability-related limitations of daily living**								
	**Low**	**Mid**				**High**			
	**OR**	**OR**	**95% CI**			**OR**	**95% CI**		
**Disability type**									
Physical or brain lesion disability	1.00	1.63	(0.97	–	2.71)	2.77	(1.22	–	6.29)
Visual, hearing or language disability	1.00	1.03	(0.60	–	1.78)	1.99	(0.62	–	6.45)
Intellectual, autism, or mental disability	-	-	-	-	-	-	-	-	-
Internal or facial disability	1.00	1.70	(0.95	–	3.05)	3.67	(0.93	–	14.47)
**Disability level**									
Low	1.00	1.59	(1.04	–	2.44)	4.58	(1.94	–	10.79)
High	1.00	1.31	(0.87	–	1.98)	2.17	(0.97	–	4.86)
**Variables**	**Unmet needs**								
	**Women**								
	**Disability-related limitations of daily living**								
	**Low**	**Mid**				**High**			
	**OR**	**OR**	**95% CI**			**OR**	**95% CI**		
**Disability type**									
Physical or brain lesion disability	1.00	1.64	(0.99	–	2.72)	0.98	(0.35	–	2.77)
Visual, hearing or language disability	1.00	1.84	(1.18	–	2.88)	1.24	(0.34	–	4.51)
Intellectual, autism, or mental disability	-	-	-	-	-	-	-	-	-
Internal or facial disability	1.00	2.04	(0.96	–	4.37)	7.45	(1.11	–	50.00)
**Disability level**									
Low	1.00	1.76	(1.19	–	2.61)	0.51	(0.07	–	3.70)
High	1.00	1.88	(1.22	–	2.91)	2.36	(1.04	–	5.36)

According to an itemized analysis of disability-related limitations in daily living in Table 5, individuals with difficulties in using their legs and feet were most at risk for having unmet health needs (men: AOR 2.35, 95% CI 1.73–3.19; women: AOR 2.68, 95% CI 2.01–3.58). The next highest risk of unmet needs was having head injuries from a bruise or fall in men (AOR 2.24, 95% CI 1.61–3.11) and pain from chronic disease in women (AOR 2.34, 95% CI 1.78–3.06).

**Table 5:** Results of unmet needs by the disability-related limitations of daily living questionnaire items

Variables	Unmet needs							
	Men				Women			
	OR	95% CI			OR	95% CI		
**Questions about limitations of daily life due to disability** ^**a**^								
I have limited vision to see things.	1.83	(1.33	–	2.52)	1.35	(0.94	–	1.96)
I have limited hearing.	1.58	(1.09	–	2.28)	1.02	(0.65	–	1.60)
I am limited in speaking.	1.01	(0.71	–	1.43)	0.94	(0.64	–	1.37)
I have a hard time breathing.	1.28	(0.90	–	1.82)	2.17	(1.58	–	2.96)
I have pain due to chronic disease.	1.63	(1.25	–	2.13)	2.34	(1.78	–	3.06)
I have limited ability to learn or understand things.	1.31	(0.97	–	1.77)	1.26	(0.94	–	1.68)
I have limited movement of my arms and fingers.	1.76	(1.27	–	2.44)	2.07	(1.50	–	2.86)
I have limitations on holding or holding something.	1.96	(1.46	–	2.62)	2.25	(1.65	–	3.08)
I have limited use of my legs and feet.	2.35	(1.73	–	3.19)	2.68	(2.01	–	3.58)
I have limited ability to control my emotions, such as anxiety and depression.	1.93	(1.46	–	2.56)	2.08	(1.58	–	2.75)
I have cosmetic damage including malformations.	1.79	(1.26	–	2.56)	1.89	(1.25	–	2.85)
I have suffered head injuries, such as hitting or falling.	2.24	(1.61	–	3.11)	1.65	(1.12	–	2.45)
I have had a stroke (paralysis).	0.94	(0.63	–	1.41)	0.65	(0.42	–	1.02)
I am limited in judging and making decisions.	1.48	(1.06	–	2.08)	1.09	(0.77	–	1.53)

^a^ OR and 95% CI values for each question are the result of meeting the unmet medical risk in the high-limitations group for the low-limitations group (reference group)

As for unmet needs by cause, men were more likely to face factors other than medical expenses in Fig. 1 (disability-related limitations in daily living, mid: AOR 2.08, 95% CI 1.06–4.05; high: AOR 7.15, 95% CI 2.72–18.78), and women were more likely to face medical expense burdens in Fig. 2 (disability-related limitations in daily living, mid: AOR 1.99, 95% CI 1.38–2.85; high: AOR 1.23, 95% CI 0.46–3.33).

## Discussion

This study found that the risk of having unmet medical care needs increased with an increase in disability-related limitations in daily living, and this effect was more pronounced in men. Men with physical, brain lesion-related, or mild disabilities, and women with internal, facial, or severe disabilities were vulnerable to having unmet health needs due to limitations in daily life. In both men and women, pain due to leg and foot limitations increased the risk of having unmet health needs. Regarding causes of unmet needs, women were more likely to have the burden of medical expenses as a cause, while men were more likely to have factors other than this as causes.

We found that the risk of having unmet needs was higher when an individual’s disabilities severely affected their activities of daily living and limited their daily life. This is similar to prior findings that unmet needs increase due to limitations in daily life caused by old age or diseases such as cancer, dementia, asthma, and osteoarthritis in previous studies [16–22]. Our results and those of previous studies, both, confirm the relationship between unmet needs and chronic or incurable diseases, including disabilities. Additionally, this study is meaningful because it is generalizable by conducting a national survey and examines the impact of disability on unmet needs based on severity, both generally and in detail.

The study found that daily limitations owing to disabilities increase the risk of having unmet needs. Women were more vulnerable to having unmet needs when their limitations in daily life were moderate. However, men were more likely to have unmet needs if their daily life limitations were severe. In previous studies, women with disabilities were found to be at a higher risk of having unmet needs than men [6, 23, 24]. Nevertheless, when care is lacking, men with disabilities are more vulnerable to unmet needs than women [25]. However, the results may have been uncertain because the absolute number of women in this study who fell into the high daily-life limitation group was small.

To examine the effect of daily life limitations on unmet needs in detail, we used the subdivisions of disability type, severity, health aspect that limits daily life, and cause of unmet needs in this study. Regarding disability type, this effect differed by sex. Men with physical or brain lesion disabilities were 2.77 times more likely to have unmet needs when their daily life limitations were higher than those with no limitations. In South Korea, physical disabilities in men were due to (1) joint injuries (60.0%), (2) amputation (19.1%), (3) paralysis (16.2%), and (4) deformity (4.6%) in 2017 [26]. Among joint injuries, 25.7% involved the spinal discs and 12.4%, the knees [26]. Additionally, one in four people with physical disabilities did not have assistance in their daily life [27]. Physical disabilities may result in gait disturbances while accessing medical institutions thereby leading to unmet health needs.

Disabilities related to brain lesions are characterized by overlapping disorders. The main comorbidities in men with brain lesions were (1) language (45.7%), (2) intellectual (24.3%), (3) visual (21.9%), and (4) hearing (17.3%) in 2017 in South Korea [26]. Among people with brain lesions, nine out of ten had helpers to execute activities of daily life. However, 44.6% respondents said that they lacked assistance from others. The reasons were (1) limited help from family (54.9%), (2) lack of time from personal assistants (23.7%), and (3) severe disability (16.0%) [26]. Brain lesions can make it difficult to obtain independent medical care because of communication or intellectual impairments. People with brain lesions may also have unmet needs owing to insufficient help from others.

Meanwhile, women with internal or facial disabilities and severe daily life limitations were 7.45 times more likely to have unmet needs. Internal disabilities include renal, cardiac, respiratory, liver, stoma, urostomy, and epileptic disabilities. External physical disability typically results from the initial cessation of the pathology caused by an injury or disease, after which disability and impairment persist. However, internal organ disabilities require continuous medical attention, including treatment, diagnosis, and testing, because disabilities and diseases exist simultaneously, and usually worsen over time [28]. In South Korea, where national health insurance is available, the average additional monthly cost due to internal disabilities was higher than the average in 2017 (average: $144.8; liver: $405.4; kidney: $257.5; stoma or urostomy $205.9) [26].

Facial disabilities had the second-highest average monthly cost of disability (at $288.8, after autism at $533.7) [26]. Additionally, people with facial disabilities were the group that most often responded that they could not visit hospitals or clinics even if they wanted to due to economic reasons [26]. The economic burden of internal or facial disabilities can create a gap between the need for and use of medical services.

Regarding limiting factors in daily life due to disability, difficulties in using legs and feet had the greatest effect on the existence of unmet needs, regardless of sex. Mobility impairment is a major concern for adults with disabilities [29, 30]. With the exception of non-face-to-face treatment and home nursing, discomfort in the legs and feet is directly related to unmet needs as physical movement becomes difficult.

The primary cause of unmet needs in women was the burden of medical expenses. Women with disabilities face a dual barrier to the labor market: being a woman and a person with disabilities [31]. The employment rate of women with disabilities was only 49.8% of that of men [26]. Considering that difficulty performing work due to disability (34.0%) and health problems (20.5%) are the main reasons for non-employment for women with disabilities who do not work, it can be concluded that limitations due to disability and unmet needs due to household difficulties are closely related.

This study has certain limitations. First, the data used in this study excluded facility residents, individuals with unknown addresses, and the deceased. However, as this accounted for 2.6% of the registered disabled persons surveyed (residence in facilities: 0.6%, expungement or death: 2.0%) [32], this would not have had a significant impact on the overall results. Second, unmet needs based on time-series changes in the degree of limitation in daily life were not analyzed. However, we can consider that disability is chronic and has a relatively stable severity. Third, we adjusted for various covariates that could affect our hypothesis testing; however, some variables were not included in the study. Congenital presence or absence of disability, acceptance of disability, and support status due to disability were not considered to be covariates. Therefore, follow-up studies that consider these factors are warranted. Fourth, it was difficult to analyze each disability type individually because of the limited number of participants. However, we attempted to differentiate disability types by categorizing them in this study.

## Conclusions

The risk of unmet needs increases as disabilities severely restrict activities of daily living. Men with high disability-related limitations of daily living had 2.79 times greater unmet needs than men with low disability-related limitations of daily living. Men are more vulnerable to unmet needs owing to limitations in daily life and due to physical or brain lesion disabilities whereas women are more likely to have unmet needs due to internal or facial disabilities. The factor that had the greatest influence on limitations in daily life due to disability was the inability in using legs or feet, and the main factor affecting unmet needs for women was medical expenses. It is thus, necessary to find ways to alleviate the unmet needs of individuals with disabilities by considering the type of disability, the aspects that restrict daily life, and the causes of unfulfillment.

## Data Availability

Public secondary data used in this study can be obtained by submitting a data application to KODDI according to the notice on the following site. https://www.koddi.or.kr/bbs/notice01_view.jsp?brdNum=7415269.

## Abbreviations

GEE: Generalized estimating equation model
AOR: Adjusted odds ratios
CI: Confidence interval
DLDP: Disability and Life Dynamics Panel
KODDI: Korea Disabled People’s Development Institute
